# Heart Failure with Mid-Range or Mildly Reduced Ejection Fraction in the Era of Sodium–Glucose Co-Transporter 2 Inhibitors: Do We Now Provide Better Care for the “Middle Child of HF”? Real-World Experience from a Single Clinical Centre

**DOI:** 10.3390/jcdd11060171

**Published:** 2024-05-31

**Authors:** Marin Viđak, Jelena Kursar, Tomislava Bodrožić Džakić Poljak, Tomislav Letilović, Jasmina Ćatić, Vanja Ivanović Mihajlović, Petra Zebić Mihić, Šime Manola, Ivana Jurin

**Affiliations:** 1 Department of Cardiovascular Medicine, Dubrava University Hospital, 10000 Zagreb, Croatia; 2 School of Medicine, University of Zagreb, 10000 Zagreb, Croatia; 3 Division of Cardiology, Department of Medicine, Merkur University Hospital, 10000 Zagreb, Croatia; 4 Faulty of Medicine Osijek, J.J. Strossmayer University of Osijek, 31000 Osijek, Croatia; 5 Department of Cardiovascular Diseases, Osijek University Hospital, 31000 Osijek, Croatia

**Keywords:** heart failure, SGLT2, HFmrEF, heart failure with mildly reduced ejection fraction

## Abstract

Heart failure (HF) with mid-range or mildly reduced ejection fraction (HFmrEF) is a separate clinical entity in the HF spectrum, with a left ventricular ejection fraction ranging from 40 to 49%. While sodium glucose co-transporter 2 inhibitors have become the cornerstone therapy for the entire HF spectrum, there are a few clinical trials of HFmrEF. This prospective observational study was conducted at Dubrava University Hospital, Zagreb, Croatia, from May 2021 to October 2023. We recruited 137 participants diagnosed with HFmrEF at admission. The majority were male, with a median age of 72 and overweight. A total of 110 participants were followed for 6 months and LVEF remained the same in the majority of patients (*n* = 62, 56.4%), improved in 32 patients (29.1%), and decreased in 3 patients (2.73%). A total of 64 participants were followed for 12 months: 39 remained the same (60.94%) and 25 improved. There were 13 deaths in (9.5%). While the empagliflozin group had a lower BMI at 6-month- and lower HbA1c at 12-month follow-up, there were no differences in death, HF hospitalizations, ER visits, or urinary tract infections in between groups. Despite recent and daily advances in the treatment of all HF phenotypes, HFmrEF still represents a challenge in everyday clinical practice.

## 1. Introduction

Heart failure (HF) with mildly reduced ejection fraction (HFmrEF) is a heterogeneous category that was first mentioned in the literature in 2014 [1] and introduced as a separate entity in the 2016 ESC HF Guidelines. In these guidelines, it was defined as heart failure with mid-range EF (HFmrEF; EF 41–49%) [2]. Extensive subsequent research has confirmed that HFmrEF has some common features with heart failure with reduced ejection fraction (HFrEF) and heart failure with preserved ejection fraction (HFpEF), but also suggests distinct differences between HFmrEF and HFrEF that warrant the term HF with ‘mildly reduced’ EF [3]. Approximately 25% of patients with HF are in the HFmrEF category at the time of presentation [4].

In the 2021 ESC Guidelines, the Task Force made no recommendations for the use of sodium–glucose co-transporter 2 inhibitors (SGLT2i) in this subgroup [5], since the evidence was based on subgroup analyses of trials that were not specifically designed to focus on HFmrEF, including trials where the overall endpoints were statistically neutral. However, the newest guidelines contained an IA recommendation on the use of SGLT2i in this subgroup [3].

The first data supporting the use of SGLT2i in patients with HFmrEF came from the SOLOIST-WHF trial, where the benefits of sotagliflozin for reducing the risk of cardiovascular death, hospitalizations, or urgent visits for HF were consistent in patients with EF < 50% or ≥50% [6]. Furthermore, the EMPEROR-Preserved trial and DELIVER trial clearly demonstrated that SGLT2i reduces the risk of cardiovascular death (CVD) or urgent visits/hospitalization for heart failure in patients with HFmrEF/HFpEF regardless of diabetes mellitus type 2 (T2D) [7,8].

The evidence base for SGLT2i’s use in HFmrEF consists of, at present, post hoc analyses, and no prospective studies have assessed the effect of SGLT2i in this subgroup of patients.

The aim of our prospective real-world study was to evaluate the use of SGLT2i in the subgroup of patients with HFmrEF.

## 2. Methods

### 2.1. Study Design and Setting

This is a prospective observational study conducted in University Hospital Dubrava, which included 137 participants with HFmrEF recruited from the local HF registry, CaRD registry (NCT06090591), from May 2021 to October 2023.

Since the intraobserver and interobserver variability of standard echocardiographic left ventricular ejection fraction (LVEF) assessment is reported to be 8–21% and 6–13%, respectively [9], we included only participants in whom EF was established by two independent echocardiographers. Following the methodology from our previously reported study, LVEF was estimated using the modified Simpson’s method [10]. During the initial assessment, there was high agreement between two echocardiographic evaluations (Kappa value 0.86). Echocardiography was performed during hospitalization, at 6- and 12-month follow-up.

All of the initial clinical, laboratory, and echocardiography findings were obtained before the introduction of SGLT2i. Patients in whom SGLT2i therapy was terminated were not excluded from the registry and the reasons for termination were collected.

The HF diagnosis was established according to the Framingham criteria [11]. Participants were included in the study if they had clinical signs and symptoms consistent with HF, echocardiographic evidence of LVEF between 41 and 49%, and elevated NT-proBNP serum levels at initial assessment (>300 pg/mL for patients without ongoing atrial flutter or fibrillation, and >600 pg/mL for patients with ongoing atrial flutter or fibrillation).

T2D was defined as glycated haemoglobin (HbA1c) ≥ 6.5% or receiving antidiabetic treatment without a time limit for the duration of the therapy. Baseline characteristics, including demographics and laboratory data at admission, were collected from medical records. The estimated glomerular filtration rate (eGFR) was calculated using the guidelines from the Chronic Kidney Disease Epidemiology Collaboration and was determined on admission and at 6-month follow-up [12].

Medical therapy was indicated and escalated according to contemporary guidelines and the discretion of the cardiologist in charge. There was no study protocol regarding medical therapy [5].

### 2.2. Participants

The registry included patients with HF, regardless of aetiology (non-ischemic/ischemic) or setting (new onset, acute-on-chronic, hospitalized; inpatient or outpatient clinic visit), in whom the therapy with SGLT2i was initiated. We did not include patients in whom SGLT2i therapy was not initiated.

### 2.3. Sample Size

Based on the main outcome measure of our study, we calculated minimal sample size using a previously published study on changes in LVEF in HFmrEF patients [13]. With the α value set to 0.05 and the power set to 0.8, we calculated that we needed 80 participants in our cohort to obtain a statistically meaningful result.

### 2.4. Outcome Measures and Data Collection

We collected sociodemographic data, body mass index, NYHA status, comorbidities, laboratory values, hospitalizations, mortality, medication and medication adherence scores, and ejection fraction values. The primary outcome was changes in EF over the follow-up time. Study endpoints were documented via telephone interviews, regular outpatient follow-up, or by electronic hospital records.

### 2.5. Ethical Considerations

The study has been approved by the Dubrava University Hospital Ethics committee (2022/1403-01).

### 2.6. Statistical Analysis

Descriptive statistics are used to present the data. Categorical variables are presented as frequencies, absolute values, or percentages, and continuous variables as mean or median values with 95% confidence intervals, depending on the distribution of the data. Comparisons were performed with Mann–Whitney or Wilcoxon’s rank sum test, categorical data were compared with the chi-squared test and expressed as numbers and percentages, and correlations were assessed using the Pearson correlation coefficient. A statistical analysis was conducted using MedCalc Statistical Software version 14.8.1 (MedCalc Software, Ostend, Belgium).

## 3. Results

### 3.1. Participants’ Characteristics

**Figure 1:**
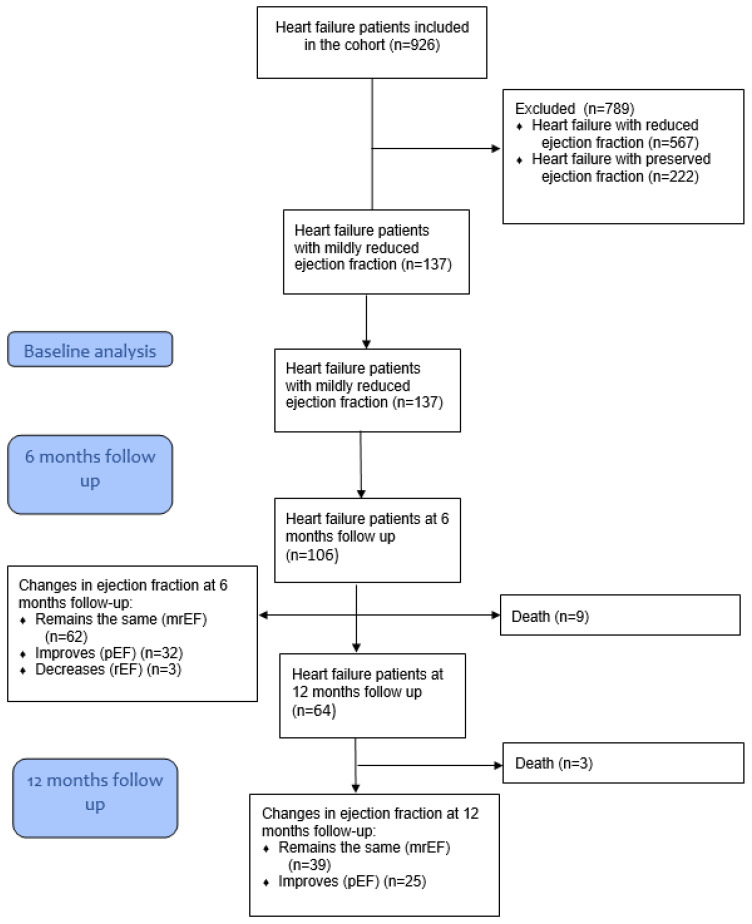
Flowchart of participants included in the study.

We recruited 137 patients diagnosed with HFmrEF at admission (Figure 1). The sociodemographic and clinical characteristics of participants are presented in Table 1. The majority were male (*n* = 92, 67.15%) with a median age of 72 (IQR 62-77). Seventy-four patients were deemed to be New York Heart Association Functional Classification (NYHA) II status (54.01%), followed by NYHA III (*n* = 50, 36.50%). The median body mass index (BMI) of the HFmrEF patients was 29 kg/m^2^ (25.5–33.33 kg/m^2^) and almost all of them had previously been diagnosed with arterial hypertension (*n* = 128, 93.43%) and dyslipidemia (*n* = 105, 76.64%). More than half of the participants had coronary artery disease that was already known at the time of recruitment (*n* = 77, 56.2%) and one third of participants had previously been diagnosed with T2D (*n* = 42, 30.66%). Thirty-four participants had peripheral artery disease (24.82%) and seventeen participants had obstructive lung disease or asthma (*n* = 17, 12.41%).

Median left ventricular ejection fraction (LVEF) at admission was 45% (interquartile range 42% to 46%); median N-terminal pro b-type natriuretic peptide (NTproBNP) levels were 1765.65 (765–4452.0).

Prior to discharge, 57 participants had percutaneous coronary intervention procedure (41.60%), 14 were transferred to cardiac surgery department for surgical coronary revascularization (10.22%), and 15 participants had a pacemaker implanted (10.95%). Thirty participants received all three traditional HF medications prior to SLGT2i initiation. Concomitant medication prior to SLGT2i initiation is presented in Table 2. Thirteen participants discontinued the use of SGLT2i, four of them due to intolerance.

### 3.2. Changes over Time

**Figure 2:**
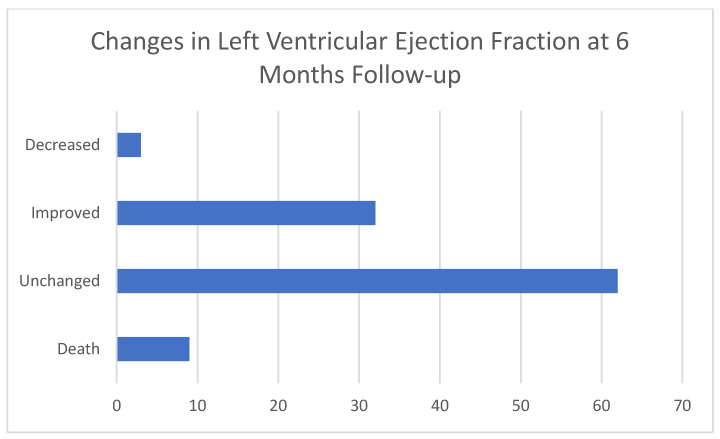
Changes in left ventricular ejection fraction at 6-month follow-up (*n* = 106).

**Figure 3:**
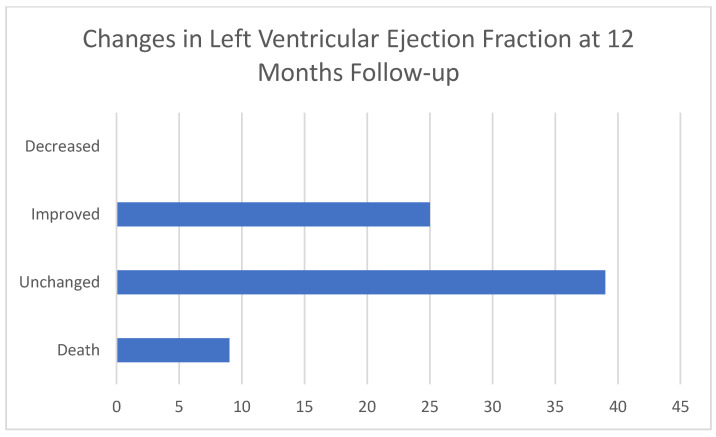
Changes in left ventricular ejection fraction at 6-month follow-up (*n* = 64).

A flowchart of participants is shown in Figure 1. We followed 106 patients for 6 months (Table 3). LVEF remained the same in the majority of patients (*n* = 62, 56.4%), improved in 32 patients (29.1%), and decreased in 3 patients (2.73%) (Figure 2). At 6-month follow-up, eGFR values remained the same (68.18 vs. 66.21, *p* = 0.1026), haemoglobin levels improved (133.52 vs. 137, *p* = 0.001), and NTproBNP levels decreased (3672.28 vs. 1776.11, *p* = 0.0002). The majority of patients’ NYHA status improved (*n* = 57, 52% were considered to be NYHA I status at 6-month follow-up). LVEF decreased in three patients. We followed 64 patients for 12 months (Table 4): 39 were still categorized as HFmrEF (60.94%), 25 improved (Figure 3), with an LVEF over 50% (39.06%), and the majority was categorized as NYHA I status at the 12-month follow-up (*n* = 36, 56.3%). During the 12-month follow-up, there was a decrease in glomerular filtration rate (68.18 vs. 48.93, *p* < 0.0001) and an improvement in both NYHA functional status and NTproBNP levels.

### 3.3. SGLT2 Inhibitor Selection and Therapy Termination

Seventy-four patients were discharged with empagliflozin (54.01%) and sixty-three with dapagliflozin (45.99%) (Table 5). There were no differences in haemoglobin, NTproBNP, and cholesterol level at 6 and 12 months between empagliflozin and dapagliflozin groups. When comparing empagliflozin and dapagliflozin, the empagliflozin group had a lower BMI at 6-month follow-up (30.99 kg/m^2^ vs. 28.95 kg/m^2^, *p* = 0.034) and lower HbA1c levels at 12 months (6.34 vs. 6.09, *p* = 0.017) (Table 6 and Table 7). SGLT2i was discontinued in 13 participants (9.5%) (Table 5).

### 3.4. Factors Contributing to LVEF Improvement and NTproBNP Levels

Lower age, higher drug adherence score, NTproBNP levels, and LVEF at 6 months were associated with recovered LVEF at 12 months (Table 8). Higher NTproBNP values were inversely correlated with a lower eGFR at both 6 months (correlation coefficient r = −0.4336, *p* < 0.0001) and 12 months (correlation coefficient r = −0.3618, *p* = 0.0031).

### 3.5. Adverse Events and Recurrent Hospitalizations

There were nine registered deaths in the first 6 months of the follow-up and three additional deaths after the 6-month assessment and prior to 12-month assessment. We registered one additional death after the 12-month assessment, with thirteen total deaths in the HFmrEF group (9.5%). The most common cause of death was worsening HF (*n* = 4), followed by acute coronary syndrome (*n* = 3), sudden cardiac death (*n* = 2), stroke (*n* = 1), sepsis (*n* = 1), and other/unknown (*n* = 2).

During the follow-up period, 44 participants were hospitalized for HF (*n* = 44, 32.11%), 13 more than once (9.49%), while 30 participants visited the emergency department for HF symptoms but were not hospitalized (21.9%). There were no differences in death, HF hospitalizations, ER visits, or urinary tract infections in the empagliflozin and dapagliflozin groups (Table 9).

## 4. Discussion

To the best of our knowledge, this was the first real-world study that recruited patients with HFmrEF in whom SGLT2i was initiated at the time of presentation. We included only patients in whom HFmrEF was the initial presentation of HF.

Considering that EF is subject to change owing to the effects of therapy or the natural progression of HF [14], we aimed to evaluate the proportion of patients with HfmrEF that might be in transition from preserved or to reduced EF as a result of an acute event.

In several previous studies that observed patients with HFmrEF, patients were younger and more likely to be male, with a higher prevalence of coronary artery disease (CAD) [15,16,17,18]. Those results are in line with our study, and although our patients’ median age was 72, 67.15% of our patients were male and 56.20% had CAD. These features suggest a resemblance between HFrEF and HFmrEF. On the other hand, the prevalence of T2D, AF, and hypertension was similar in HFpEF and HFmrEF [15,16,17,18]. In our study, previously diagnosed T2D was present in 30.66% of patients, and in 8.76% patients, T2D was newly diagnosed during hospital stay. We found a very high prevalence of arterial hypertension in our cohort, with 93.43% of patients having diagnosed arterial hypertension. AF was present in 45.2% of participants, with the majority being permanent AF (10.95% of our patients had paroxysmal AF, 11.68% of our patients had persistent AF, and 22.63% had permanent AF). The diagnosis of AF was documented with an electrocardiogram (ECG) tracing showing AF for a duration of a least 30 s [19].

As observed in our previous study [10], HFmrEF patients more frequently had permanent AF, and patients with AF and HFmrEF are a special subgroup of patients presenting with distinct clinical characteristics and an increased risk of stroke/systemic embolism and death. AF is considered to be an important aspect of HFpEF [20], as well as a risk factor in mortality and HF hospitalizations in HFmrEF patients [21]. In some HFpEF subgroups, AF is often the first presentation of the disease, causing the initial decline in LVEF (sometimes diagnosed as HFmrEF), and treating AF improves LVEF in one third of patients [22].

HF patients often present with chronic kidney disease (CKD) and a deterioration of kidney function. While NTproBNP levels are considered an important biomarker of cardiac function and can help in diagnosing HF [23], our study shows that decreased renal function is inversely correlated with NTproBNP levels, and future studies are needed to determine more precise cut-off points when assessing patients with both HF and CKD.

There were 13 deaths (9.48%) during follow-up. The most common reported cause of death was heart failure, followed by acute myocardial infarction and stroke. These observations highlight the fact that patients with HFmrEF are a vulnerable group that require more intensive control and a specific therapeutic approach. The newest ESC HF Guidelines contained an IA recommendation of the use of SGLT2i in patients with HF, presenting SGLT2i as a “game changer” drug across the HF continuum [3]. All of our patients were discharged with SGLT2i, but 9.48% of patients discontinued the therapy during follow-up. To the best of our knowledge, no previous studies have addressed the reasons for the discontinuation of SGLT2i in patients with HFmrEF. However, a recent study [22] has addressed the reasons for the discontinuation of SGLt2i in patients with T2D. The most common reason for discontinuing SGLT2is was frequent urination (19.6%), followed by urogenital infections (11.3%). The most common reported reason for drug discontinuation in our cohort was intolerance to drugs (self-reported by patients and including dizziness, nausea, or a rash). The second most prevalent reason was patients’ own decision to discontinue the drug based on their self-assessment, with no known side effects being reported (“patient thinks that they do not need the drug”). We can only assume that, considering the fact that SGLT2 drug costs are not fully covered by universal healthcare insurance in our country, part of this reasoning is financial in nature. However, health literacy and physician–patient communication may also play a part. Considering that this reason is not negligible, it is up to us to work on improving communication with patients as well as improving their health literacy. It is known that effective and clear physician–patient communication is imperative to achieve and maintain the best therapeutic benefits [24]. It is also important to campaign for our patients by ensuring that health insurance follows up on the relevant clinical practice guidelines. As mentioned above, in our country, the costs of SGLT2i are not fully covered and patients have to participate in the costs. SGLT2i are the only drugs that have an IA recommendation for the treatment of HFpEF and HFmrEF; therefore, efforts are needed to minimize treatment costs and to ensure that all patients receive optimal care regardless of their financial status. As previously reported, HFmrEF represents a large group of patients with heterogenous features and consist of at least three subgroups, including HFmrEF improved, HFmrEF unchanged, and HFmrEF deteriorated [17,18,25]. The majority of patients in our cohort, although significantly improved in NYHA status, were in the subgroup „HFmrEF unchanged” and a minority were in the subgroup “HFmrEF deteriorated”.

In our study, we identified several factors associated with an improvement in ejection fraction after 12-month follow-up. While older age is a known risk factor for deteriorating heart function, this is the first study, to our knowledge, to assess the impact of drug adherence on EF improvement and the first one to assess the adherence to SGLT2i among patients with HFmrEF.

SGLT2i is the only medication that is shown to be effective over the entire HF spectrum. Despite the myriad of cardioprotective benefits, the exact mechanism by which SGLT2i reduces the burden of the disease is still not known. SGLT2i has beneficial effects in the control of blood pressure, glucose homeostasis, and body mass index, all of which modify patients’ cardiovascular risk profile [26]. Considering that most patients in our cohort had CAD, we can attribute the possible beneficial effect of SGLT2i to this mechanism. Also, SGLT2i increases coronary flow, predominantly by improving vascular function and reducing sympathetic tone. As previously mentioned, these features suggest a resemblance between HFrEF and HFmrEF [27].

However, the prevalence of T2D, AF, and hypertension is similar in HFpEF and HFmrEF. Potential mechanisms that would work favourably in this group, in addition to glucose homeostasis and pressure control, could be the reduction in fibrosis of both the ventricles and the atria and the consequent improvement in diastolic function leading to positive remodelling.

Large clinical trials and meta-analyses have investigated the role of SGLT2 inhibition in incident AF, but the results remain controversial [28,29]. A recent study has suggested that the treatment effects of SGLT2i were associated with a lower incidence of HF hospitalization in patients with AF [30].

Moreover, SGLT2i promotes natriuresis secondary to glycosuria, which reduces preload and consequently improves cardiac function in patients with HF. Unlike conventional diuretics, SGLT2 inhibition differentially regulates interstitial fluid volumes, and therefore does not produce a noxious, overstimulated, sympathetic response in HF patients. At a molecular level, SGLT2i decreases oxidative stress and inflammation, prevents cardiomyocyte cell death, improves cardiac energy metabolism, prevents extracellular matrix remodelling and cardiac fibrosis, decreases epicardial fat, and promotes endothelial function [31]. Given the plethora of effects of SGLT2i in patients with HF, and the heterogeneity of the HFmrEF, future studies are needed to determine the dominant mechanism of action in different phenotypes of HFmrEF.

### Strengths and Limitations

The present study has some limitations. First, it is based on data from an observational registry. Due to the character of the registry (eligibility depending on initiation of SGLT2i therapy), patients with contraindications for SGLT2i were omitted (e.g., type 1 diabetes, frequent urinary infections). We did not include HFmrEF patients in whom the SGLT2i therapy was not initiated and no comparison with SGLT2i-naïve patients was made. In addition, inclusion in the registry was allowed for the whole spectrum of HFmrEF patients (hospitalised vs. outpatient; acute vs. stable HF; ischemic vs. non-ischemic), a fact inevitably related to the population’s heterogeneity. Secondly, this observational registry was not followed-up with a detailed study protocol, and due to its nature, the intervention allocation, i.e., SGLT2i initiation, was entirely dependent on the clinicians’ decision. However, we believe this is also a potential strength of our study, because it provides insight into real-life practice, similarly to pragmatic design studies.

Thirdly, the study was conducted in a tertiary centre with experienced HF specialists, which could represent a bias since real-world data should also reflect the treatment of patients in hospitals that do not have dedicated HF specialists.

## 5. Conclusions

Despite recent and daily advances in the treatment of all HF phenotypes, HFmrEF still represents a challenge in everyday clinical practice. Although our results suggest that HFmrEF could be a unique phenotype, this issue is still controversial, as some patients still transition to HFpEF and HFrEF (HFmrEF improved and deteriorated).

The so-called “middle child of HF” [1] is poorly represented in clinical research, with no appropriate RCTs. Because of this, in clinical practice, HFmrEF patients are frequently medicated with standard HF therapy due to concomitant indications [18].

More prospective and robust studies are needed to better understand the underlying causes of HFmrEF and to reach a consensus on the HFmrEF diagnostic criteria, as well as to help tailor the development of targeted interventions to improve the management of HFmrEF.

## Figures and Tables

**Table 1:** Sociodemographic data and clinical characteristics of the patients with heart failure with mildly reduced ejection fraction (*n* = 137).

Category	*n*	%
Gender		
Male	92	67.15%
Female	45	32.85%
Age (C, IQR)	72 (62–77)	
NYHA status		
NYHA 1	9	6.57%
NYHA 2	74	54.01%
NYHA 3	50	36.50%
NYHA 4	4	2.92%
Atrial fibrillation/flutter (total)	62	45.25%
Paroxysmal	15	10.95%
Persistent	16	11.68%
Permanent	31	22.63%
BMI (C, IQR)	29 (25.5–33.3)	
Comorbidities		
Coronary artery disease	77	56.20%
Dyslipidaemia	105	76.64%
Stroke/TIA	10	7.30%
Peripheral art disease	34	24.82%
COPD/asthma	17	12.41%
HA	128	93.43%
Smoking	47	34.31%
Diabetes		
Yes	42	30.66%
Discovered during hospital stay	12	8.76%
Prediabetes	34	24.82%
LVEF (C, IQR)	45 (42 to 46)	
Serum values (C, IQR)		
NT-proBNP (pg/mL)	1764.5 (765.0–4452.0)	
eGFR (mL/min/1.73 m^2^)	66.6 (47.4–83.1)	
Total cholesterol (mmol/L)	4.3 (3.6–5.3)	
LDL (mmol/L)	2.4 (1.8–3.3)	
HDL (mmol/L)	1.1 (0.9–1.4)	
Total triglycerides (mmol/L)	1.4 (1.1–1.9)	
HbA1c (%)	6.2 (5.6–6.9)	
Potassium (mmol/L)	4.4 (4.1–4.6)	
Sodium (mmol/L)	139 (138–141)	
Chloride (mmol/L)	103 (100–105)	
Adherence score (C, IQR)	7 (5.25–8)	
HF OMT at presentation	30	21.90%

C—median; IQR—interquartile range; NYHA—New York Heart Association Functional Classification; BMI—body mass index; TIA—transient ischemic attack; COPD—chronic obstructive pulmonary disease; LVEF—left ventricular ejection fraction; eGFR—estimated glomerular filtration rate; NT-proBNP—N-terminal prohormone of brain natriuretic peptide; LDL—low-density lipoprotein; HLD—high-density lipoprotein; HbA1c—haemoglobin A1C.

**Table 2:** Concomitant medication prior to and after sodium–glucose-transporter-2 inhibitors initiation (*n* = 137).

	Prior to SGLT2i Initiation		After SGLT2i Initiation	
Drug	Number	%	Number	%
Angiotensine converting enzyme inhibitors (any)	81	59.12	105	76.64
Ramipril	30	21.90	30	21.90
Perindopril	46	33.58	71	51.82
Zofenopril	4	2.92	3	2.19
Enalapril	1	0.73	-	
Lizinopril	-		1	0.73
Angiotensin receptor blockers (any)	10	7.30	13	9.49
Valsartan	7	5.11	8	5.84
Losartan	3	2.19	5	3.65
Mineralocorticoid receptor antagonist (any)	52	37.96	110	80.29
Eplerenone	47	34.31	107	78.10
Spironolactone	5	3.65	3	2.19
Beta blockers (any)	65	47.45	118	86.13
Bisoprolol	52	37.96	94	68.61
Metoprolol	4	2.92	10	7.30
Carvedilol	3	2.19	5	3.65
Nebivolol	6	4.38	8	5.84
Statin (any)	52	37.96	106	77.37
Angiotensin receptor/neprilysin inhibitor	9	6.57	11	8.03
Other diuretics (any)	69	50.36	94	68.61
Furosemide	53	38.69	81	59.12
Torsemide	3	2.19	5	3.65
Indapamide	7	5.11	6	4.38
Hydrochlorothiazide	5	3.65	1	0.73
Calcium channel blockers (any)	28	20.44	31	22.63
Amiodarone	5	3.65	5	3.65
Digoxin	4	2.92	8	5.84
Metformin	16	11.68	33	24.09
GLP 1	1	0.7	10	7.30
Another oral hypoglycaemic drug	9	6.6	5	3.65
Insulin	5	3.65	9	6.57

**Table 3:** Six-month follow-up of patients with heart failure with mildly reduced ejection fraction (*n* = 106).

Variable (C, IQR)	Baseline	6-Month Follow-Up	*p*-Value *
eGFR (mL/min/1.73 m^2^)	68.18 (64.05–72.3)	66.21 (61.84–70.59)	0.1026
Haemoglobin (g/L)	133.52 (130.04–135.99)	137 (134.18–141.78)	0.001
NTproBNP (pg/mL)	3672.28 (2334.98–5009.57)	1776.11 (994.92–2557.3)	0.0002
LVEF (%)	44.6 (44.3–44.9)	47.1 (45.83–48.35)	0.0001
NYHA status	2 (2–3)	1 (1–2)	<0.0001

C—median; IQR—interquartile range; eGFR—estimated glomerular filtration rate; NT-proBNP—N-terminal prohormone of brain natriuretic peptide; LVEF—left ventricular ejection fraction; NYHA—New York Heart Association Functional Classification. * Wilcoxon paired sample test.

**Table 4:** Twelve-month follow-up of patients with heart failure with mildly reduced ejection fraction (*n* = 64).

Variable (C, IQR)	Baseline	12-Month Follow-Up	*p*-Value *
eGFR (mL/min/1.73 m^2^)	68.27 (62.67–73.86)	48.93 (47.1–50.78)	<0.0001
Haemoglobin (g/L)	133.48 (128.77–138.19)	136.71 (132.42–141)	0.072
NTproBNP (pg/mL)	3232.87 (1895.13–4570.59)	1583.11 (971.1–2195.12)	0.0015
LVEF (%)	44.58 (44.3–45.9)	48.9 (47–50.8)	<0.0001
NYHA status	2 (2–3)	1 (1–2)	<0.0001

C—median; IQR—interquartile range; eGFR—estimated glomerular filtration rate; NT-proBNP—N-terminal prohormone of brain natriuretic peptide; LVEF—left ventricular ejection fraction; NYHA—New York Heart Association Functional Classification. * Wilcoxon paired sample test.

**Table 5:** Sodium–glucose co-transporter 2 inhibitors drug at discharge and reasons for discontinuation of the drug (*n* = 137).

Sodium–Glucose Co-Transporter 2 Inhibitors at Discharge	Number (%)
Dapagliflozin	63 (45.99)
Empagliflozin	74 (54.01)
Switched at follow-up	1 (0.7)
Discontinued	13 (9.5%)
Reasons:	
Intolerance	5
Patient discontinued due to financial reasons	1
Urinary tract infection	1
Patient discontinued with no reasons provided	6

**Table 6:** Comparison of empagliflozin and dapagliflozin in patients with mildly reduced ejection fraction at 6-month follow-up (*n* = 106).

Laboratory Value (C, IQR)	Dapagliflozin (*n* = 47)	Empagliflozin (*n* = 59)	*p*-Value *
eGFR (mL/min/1.73 m^2^)	64.23 (55.53–72.93)	67.4 (60.92–73.88)	0.064
Haemoglobin (g/L)	140.34 (132.49–148.19)	134.1 (127.21–140.91	0.451
NTproBNP (pg/mL)	1586.69 (861.53–2311.84)	1408.21 (714.34–2102.1)	0.946
NYHA status	2 (1–2)	1 (1–2)	0.3173
BMI (kg/m^2^)	30.99 (28.81–33.18)	28.95 (27.46–30.44)	0.034
HbA1c (%)	6.46 (6.13–6.79)	6.03 (5.78–6.28)	0.169
HDL (mmol/L)	1.29 (1.17–1.41)	1.23 (1.12–133)	0.892
LDL (mmol/L)	2.26 (1.92–2.59)	2.06 (1.82–2.3)	0.157
Total cholesterol (mmol/L)	4.34 (3.87–4.81)	3.86 (3.53–4.17)	0.292

C—median; IQR—interquartile range; eGFR—estimated glomerular filtration rate; NT-proBNP—N-terminal prohormone of brain natriuretic peptide; LVEF—left ventricular ejection fraction; BMI—body mass index; NYHA—New York Heart Association Functional Classification; HbA1c—haemoglobin A1C; LDL—low-density lipoprotein; HLD—high-density lipoprotein. * Mann–Whitney test.

**Table 7:** Comparison of empagliflozin and dapagliflozin in patients with mildly reduced ejection fraction at 12-month follow-up (*n* = 64).

Laboratory Value (C, IQR)	Dapagliflozin (*n* = 33)	Empagliflozin (*n* = 31)	*p*-Value
eGFR (mL/min/1.73 m^2^)	64.62 (56.63–72.65)	69.46 (63–75.91)	0.222
Haemoglobin (g/L)	138.11 (131.95–144.27)	135.18 (128.92–141.44)	0.0867
NTproBNP (pg/mL)	1406.51 (691.41–2121.62)	1776.28 (720.45–283.11)	0.054
NYHA status	2 (1–2)	1 (1–2)	0.6925
BMI (kg/m^2^)	31.7 (29.97–34.45)	28.83 (26.53–21.12)	0.133
HbA1c (%)	6.43 (6.02–6.84)	6.09 (5.81–6.37)	0.017
HDL (mmol/L)	1.32 (1.16–1.48)	1.28 (1.1–1.46)	0.557
LDL (mmol/L)	2.19 (1.84–2.55)	2.06 (1.74–2.38)	0.522
Total cholesterol (mmol/L)	4.068 (3.51–4.62)	3.86 (3.54–4.17)	0.404

**Table 8:** Comparison of characteristics of the group of participants with improvement in ejection fraction at 12-month follow-up and the group without improvement (*n* = 64).

Variable (C, IQR)	Improvement Group	Non-Improvement Group	*p*-Value *
Adherence score	8 (7–8)	6.375 (5.75–7.341)	0.0131
BMI (kg/m^2^)	29.3 (26.459–32.76)	29.2 (27.27–31.18)	0.8311
Age	69 (61.15–72.85)	73 (67–76)	0.0459
LVEF at 6 months (%)	50 (45.0–51.7)	45 (44–46)	<0.0001
LVEF at admission (%)	44. (44.35–45.25)	44.44 (44.05–44.82)	0.633
NTproBNP levels at admission (pg/mL)	1387.0 (666.15–1965.39)	1796.0 (1191.73–2596.58)	0.1888
NTproBNP levels at 6 months (pg/mL)	391.5 (260.99–621.88)	987.0 (865.85–1379.81)	0.0003
eGFR at admission mL/min/1.73 m^2^)	67.3 (60.78–87.71)	70.2 (55.96–81.12)	0.6301
eGFR at 6 months mL/min/1.73 m^2^)	77.5 (58.48–88.0)	62.75 (50.4–74.1)	0.1328
Haemoglobin at admission (g/L)	135 (128.15–139.85)	140 (121.92–144)	0.9671
Haemoglobin at 6 months (g/L)	140 (136.7–146.65)	138 (125–145)	0.6505

C—median; IQR—interquartile range; BMI—body mass index; eGFR—estimated glomerular filtration rate; BMI—body mass index; NT-proBNP—N-terminal prohormone of brain natriuretic peptide. * Mann–Whitney test.

**Table 9:** Comparison of total deaths, heart failure hospitalization, heart failure emergency department visits, and urinary tract infections in patients with heart failure with mildly reduced ejection fraction treated with dapagliflozin and empagliflozin (*n* = 137).

	*n* (%)			*p*-Value *
	Total (*n* = 137)	Dapagliflozin (*n* = 63)	Empagliflozin (*n* = 74)	
Deaths	13 (9.5)	8 (12.7)	5 (6.8)	0.4449
HF hospitalizations	44 (32.12)	19 (30.16)	25 (33.78)	0.878
HF ED visits	30 (21.9)	14 (22.22)	16 (21.62)	0.068
UTI	13 (9.49)	8 (5.9)	5 (3.65)	0.2856

HF—heart failure; ER—emergency department; UTI—urinary tract infection. * Chi-squared test.

## Data Availability

Data will be available upon request.
